# When Protein Structure Embedding Meets Large Language Models

**DOI:** 10.3390/genes15010025

**Published:** 2023-12-23

**Authors:** Sarwan Ali, Prakash Chourasia, Murray Patterson

**Affiliations:** Department of Computer Science, Georgia State University, Atlanta, GA 30303, USA; sali85@student.gsu.edu (S.A.); pchourasia1@student.gsu.edu (P.C.)

**Keywords:** PDB files, protein structure, classification, representation learning, LLM

## Abstract

Protein structure analysis is essential in various bioinformatics domains such as drug discovery, disease diagnosis, and evolutionary studies. Within structural biology, the classification of protein structures is pivotal, employing machine learning algorithms to categorize structures based on data from databases like the Protein Data Bank (PDB). To predict protein functions, embeddings based on protein sequences have been employed. Creating numerical embeddings that preserve vital information while considering protein structure and sequence presents several challenges. The existing literature lacks a comprehensive and effective approach that combines structural and sequence-based features to achieve efficient protein classification. While large language models (LLMs) have exhibited promising outcomes for protein function prediction, their focus primarily lies on protein sequences, disregarding the 3D structures of proteins. The quality of embeddings heavily relies on how well the geometry of the embedding space aligns with the underlying data structure, posing a critical research question. Traditionally, Euclidean space has served as a widely utilized framework for embeddings. In this study, we propose a novel method for designing numerical embeddings in Euclidean space for proteins by leveraging 3D structure information, specifically employing the concept of contact maps. These embeddings are synergistically combined with features extracted from LLMs and traditional feature engineering techniques to enhance the performance of embeddings in supervised protein analysis. Experimental results on benchmark datasets, including PDB Bind and STCRDAB, demonstrate the superior performance of the proposed method for protein function prediction.

## 1. Introduction

Supervised analysis for proteins is a well-established field in bioinformatics and biochemistry, focusing on the relationship between sequence, structure, and function. While protein sequences have traditionally been the main input for classification [1], modern approaches incorporate additional data such as secondary structure, solvent accessibility, disorder propensity, and multiple sequence alignments (MSAs). Understanding protein features and properties is crucial for comprehending their function and interactions. Databases like the Protein Data Bank (PDB) provide valuable resources of protein structural information, facilitating detailed analysis and exploration.

Analysis of proteins is a prominent research field within computational biology, offering numerous applications such as enzyme design [2], protein–protein interactions [3,4], and facilitating drug discovery strategies [5]. The Protein Data Bank (PDB) [6] has played a pivotal role in providing a vast repository of protein structures, enabling comprehensive studies on protein structure and function. Protein function prediction helps in understanding biological processes, finding new drugs, treating illnesses, and many more applications [7,8,9]. It all depends on our ability to predict the actions of proteins. When studying protein function, amino acids from protein sequences look random, but they exhibit a pattern and are not random [10]. Thus, they are primarily helpful for understanding its function, and thus several sequence-based approaches are popular [11,12]. This covers methods such as motif/domain identification [13], homology modeling [14,15], and sequence alignment [16]. Another approach that is frequently employed in protein analysis is a structure-based methodology, which looks at the three-dimensional structure of the protein to infer its function [17]. This entails methods including docking simulations [18], structure–function connection analysis, and protein structure modeling [19]. Researchers have recently begun to use machine learning in bioinformatics to analyze large datasets, extract patterns, and predict protein functions based on known features, such as sequence, structure, or functional annotations [20,21]. This approach makes use of statistical models and computational algorithms. The complexity and diversity of protein functions, along with the ongoing discovery of new proteins with unique functions, make this an area of great challenge. Research is still being conducted to increase prediction accuracy and learn more about how proteins function in biological systems [22,23]. A greater comprehension of proteins and their functions in biological processes can be attained by considering both the protein sequence and its associated structure. Using an integrated approach, we can better investigate and understand the complexities of protein biology.

To predict a protein function, information from its structure or sequence can be extracted with the aid of language models [18,24,25]. These models have the potential to reveal patterns or relationships that are difficult to identify using more conventional techniques by examining the contextual information contained in sequences or structures [26]. Language models do not have direct access to the intricate three-dimensional structures of proteins; instead, they rely on textual data. Protein activity frequently relies on complex structural information that cannot be fully understood from textual data alone [14]. Because proteins have different structures and activities, it is difficult for language models to represent the subtleties and details of each protein’s operation using just textual patterns [27]. Furthermore, despite their superior ability to comprehend language patterns, language models may not possess the biological background necessary to comprehend the context of protein function. Accurate prediction requires an understanding of biological interactions, processes, and metabolic pathways. It may be difficult to appropriately understand and confirm predictions made by language models since they may not be born with this domain-specific knowledge [28]. Large language models exhibit potential across multiple domains. Predicting the function of proteins is a challenging issue involving multiple interdisciplinary domains, including structural biology, molecular biology, and bioinformatics [29]. While language-centric AI models can help with data analysis and pattern recognition, a thorough understanding of protein interactions, structures, and functions frequently necessitates specific training in these scientific fields. Accurate and trustworthy predictions in this area still depend on integrating knowledge from several domains [30]. Achieving precise and dependable protein function prediction still requires integrating massive language models with domain-specific information and experimental confirmation.

Protein classification has shifted from knowledge-based statistical reasoning (involves pre-existing knowledge, domain expertise) to the integration of machine learning techniques (data-driven approaches), including neural networks [31,32,33] and SVMs [34]. Recent studies have explored both alignment-based [35] and alignment-free [36,37] methods for protein sequence analysis. However, sequence-only approaches like SeqVec [37] and ProteinBert [38] have limitations in generalization due to the complexity of protein sequences. It is crucial to incorporate structural information and other sequence properties to overcome these limitations, enabling the development of robust and practical protein classification methods. Proteins, composed of amino acids or polypeptides, serve as essential building blocks in biological systems. The primary structure represents the linear arrangement of amino acids, while the secondary structure describes local folding patterns such as beta-pleated sheets and alpha helices along the polypeptide backbone [39]. The tertiary structure encompasses the overall three-dimensional arrangement achieved through the folding of the polypeptide chain. Even minor changes in the primary structure can significantly impact the protein’s structure and function, underscoring the importance of comprehending biomolecular structure in various health- and disease-related contexts.

The contact map-based embedding design utilizes the three-dimensional (3D) structure of proteins to create numerical representations. The contact map is a method that encodes the spatial proximity between amino acid residues in a protein. By leveraging the information from the contact map, the proposed method constructs embeddings that capture the structural characteristics of proteins. This approach takes into account the physical interactions and folding patterns of the protein, providing a more comprehensive representation compared to sequence-based embeddings. By incorporating the 3D structure of proteins, the contact map-based embedding design enhances the ability to capture crucial structural features and enables more accurate protein classification and function prediction. Our contributions in this paper are as follows:

1. We propose a contact map-based method to encode the 3D protein structure into a fixed-dimensional numerical representation, which can be used for efficient protein function prediction.
2. We incorporate extra features within our contact map-based embeddings using the features extracted from large language models for protein sequences, which enhance the overall predictive performance of the proposed model.
3. We also incorporate the features designed from protein sequences within our 3D structure-based embeddings to further improve the classification accuracy for protein function prediction.
4. The in-depth analysis of the proposed embedding model on two benchmark datasets shows superior predictive performance for the proposed method compared to recent baselines.

The organization of the manuscript is as follows: Section 2 provides a previous research overview, Section 3 discusses the proposed approach, Section 4 shows the experimental setup, Section 5 presents the results, and Section 6 concludes the paper.

## 2. Related Work

The study of biological sequences is a popular area of study in science. Understanding the behavior, functions, and interactions of proteins within biological systems is crucial for determining the functional and structural characteristics of the protein. Protein analysis [40] reveals information about how it interacts with other molecules and functions in different pathways and its potential associations with diseases. Moreover, understanding the structural characteristics of proteins aids in comprehending their functional roles, as structure often dictates function in biology [17,19]. Protein function and structure prediction is an essential component of biomedical research since it allows scientists to understand their mechanisms, create targeted therapies, and create treatments for a wide range of diseases [2]. Protein analyses can aid in the understanding of diseases and the development of preventative measures like drug discovery [5,41].

Traditionally, these modules relied on a mixture of physics-based energy functions, knowledge-based statistical reasoning, and heuristic algorithms [42,43], such as homology-based methods [14,15], which look up homologous sequences in a database of sequences. Every day, new sequences of amino acids and nucleotides are added to publicly available international databases, increasing the likelihood of discovering meaningful homologies. These databases can be searched for close homologs using a variety of tools and techniques like BLAST [44,45], all of which calculate sequence similarity to uncover significant biological relationships. However, researchers have been using an infusion of machine learning for over a decade. Work on protein structural classification has been ongoing for over a decade using supervised ML algorithms, such as neural networks [31,32,33] and support vector machines (SVMs) [34].

For biological sequence analyses, several feature engineering-centric approaches have been presented. Among them is OHE [35], which offers a straightforward mechanism for mapping the sequences into numerical vectors. For machine learning (ML) tasks like classification and clustering, some alignment-based [35,46] and alignment-free [47] embedding techniques have gained popularity. These methods do, however, also have scaling problems because of the extraordinarily high dimensionality of feature vectors. In metagenomics, the *k*-mers-based approach is also used for sequence analysis [36,48], but their inherent sparsity limits their usefulness.

For metagenomic data, the authors of [49] recommend using minimizers. Because metagenomic data contain short reads, only one minimizer (*m*-mer) can fully describe the data. However, all these methods only consider the basic structure of the protein, which is only the arrangement of its amino acids, without accounting for the three-dimensional form of the protein. A protein’s structure contains a multitude of physiochemical properties that are not fully explored in the literature. The proteins that make up multiple sequence alignments (MSAs) are related to each other evolutionarily for every structure, and they can be a crucial source of evolutionary information for contemporary protein structure prediction [50]. However, creating MSAs can be a computationally expensive process. In the literature, kernel-based techniques for sequence classification have also been proposed [51]. However, their memory consumption is high and the biggest drawback for all these methods is that they do not have biochemical features incorporated in them and are focused on sequences only. Although SeqVec is effective at describing and encoding biochemical features, it cannot infer crucial information about, for example, the activities of proteins [52].

## 3. Proposed Approach

In this section, we begin by giving a high-level overview of the proposed approach and then discuss in detail the process of extracting sequences from Protein Data Bank (PDB) files, followed by a discussion of the embedding method.

**Figure 1:**
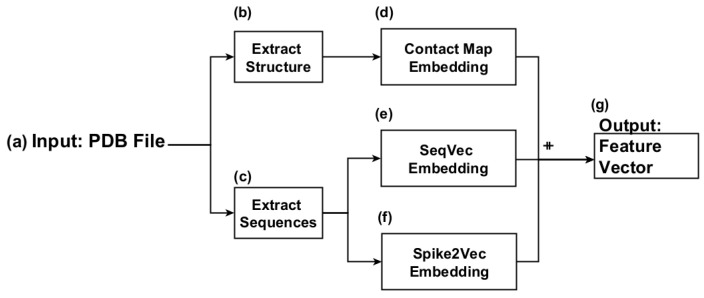
Workflow for proposed approach. We provide PDB files as input in (**a**). Then Extract the structural information (**b**). In parallel, we extract the sequences from these files (**c**). Contact Map embedding is generated using the structural information (**d**). Whereas sequences are used to generate SeqVec embeddings (**e**) and Spike2Vec embeddings (**f**). Finally, we use a combination of these embeddings generated by concatenating them to generate Feature vectors (**g**).

Figure 1 shows a high-level overview of the proposed approach. The PDB file is used as input to extract the sequences and structural information, as shown in Figure 1a–c. Using the structural information, we generate contact map-based embeddings, as shown in Figure 1d, whereas extracted sequences are used to generate LLM-based SeqVec embeddings and Spike2Vec embedding, as shown in Figure 1e,f. We evaluate these embeddings and their concatenated combinations to generate a feature vector to provide input for machine learning classifiers. Each step is discussed in detail below.

### 3.1. Sequence Extraction

Given the vital functions that proteins play in a wide range of scientific disciplines, we must comprehend the structure and function of proteins. To decipher protein sequences, we leverage the Protein Data Bank (PDB), a large data repository that embodies the complex three-dimensional structures of proteins. The methodical parsing of PDB files, which identifies the alpha-carbon atoms that comprise the fundamental protein backbone, is a necessary step in the extraction process. Afterward, this technique uses one-letter codes to stand in for the matching residues of amino acids. The result of this methodical process produces brief but meaningful sequences that elegantly capture the complex subtleties of protein structures, which enable in-depth examinations and large-scale research projects across numerous scientific fields. This basic knowledge drives advances and breakthroughs in a wide range of scientific fields, including medicine, biochemistry, structural biology, and beyond. We thus obtain concise and comprehensible sequences that represent the protein structure. To carry out the extraction procedure, a specialized tool called a PDB parser is utilized, which functions to carefully traverse and extract the relevant information contained within the intricate PDB files. This parser carefully focuses on the alpha-carbon atom connected to every amino acid residue, which is an essential step in defining the basic structure of proteins. This separation of the backbone is important because it serves as a structural framework that makes it possible to identify the complex spatial arrangement of amino acids that make up the architecture of the protein. After this important separation, the amino acid residues are methodically mapped to their respective one-letter codes using a mapping process. This mapping is usually achieved by using a dictionary, which is a tool that associates each residue of an amino acid with its assigned one-letter representation. The result of this methodical process is a concise but comprehensive representation of the protein sequence, which condenses the complicated structural features of the protein into a manageable form. This simplified form simplifies the comprehension and exchange of intricate protein structures, enabling in-depth examinations and thorough research in a variety of scientific fields. This intricate extraction procedure is an essential first step towards unraveling the complex world of proteins and advancing our understanding of their structural complexity, biological systems, and their functional importance.

One-letter codes are widely employed in molecular biology as a means to represent amino acid sequences. Each code corresponds to a specific amino acid, facilitating rapid and convenient identification of protein sequences by researchers. This process of sequence extraction is applied to all PDB files, generating a comprehensive collection of sequences that can be utilized for subsequent analysis. The extraction and analysis of protein sequences offer valuable insights into the structure and function of proteins, with implications spanning diverse fields including medicine, biochemistry, and biotechnology. By exploring these sequences, researchers can gain a deeper understanding of protein properties, enabling advancements in various scientific disciplines.

### 3.2. Contact Map-Based Embedding Generation

To generate the embeddings from the protein structures, we use the idea of a contact map. The algorithmic pseudocode for generating the embeddings from PDB files is given in Algorithm 1. The main goal of the approach is to create a protein embedding representation that works by using contact maps that are based on the spatial correlations between C-alpha atoms. This complex procedure begins with the extraction of relevant structural information from the given Protein Data Bank (PDB) file, with a particular emphasis on locating the spatial locations of the C-alpha atoms. Once these crucial coordinates are obtained, the method uses them to determine the pairwise distances between every pair of C-alpha atoms. After this computation, a distance matrix representing the subtle distances between each pair of atoms in the protein structure is produced. Applying a threshold distance, a configurable hyperparameter is a crucial step that follows to aid in the delineation of meaningful atom interactions. This criterion serves as a discriminating factor, distinguishing distances that fall below the specified threshold from those that cross it. By carefully using this thresholding method, the contact map—a visual depiction that separates atom pairs according to their spatial proximity—is produced. A spatial proximity or contact between the corresponding C-alpha atom pair is indicated by the element in the contact map that represents a distance that is less than the given threshold, marked as 1. Elements with distances greater than the threshold, on the other hand, are denoted with a 0, indicating that there is no physical contact or spatial proximity between the respective atom pairs. Using this advanced method, not only is the structural information contained in the atomic arrangement of the protein retrieved but an informative contact map is produced, which accurately depicts the critical spatial interactions between C-alpha atoms. The protein embedding representation becomes more flexible and adaptable as a result of the ability to define significant atom interactions with flexibility through adjustment of the threshold distance parameter.
**Algorithm 1** Generating Contact Map-Based Embedding1:**function** GenerateContactMap(pdb_file)2:    structure←getStructure(“protein”,pdb_file)3:    model←getPolyPeptideChain(structure)4:    c_alpha_atoms←[]5:    **for each** chain **in** model **do**6:        **for each** residue **in** chain **do**7:           c_alpha_atoms.append(residue[“CA”].get_coord())8:        **end for**9:    **end for**10:    n_atoms←len(c_alpha_atoms)11:    distances←initializeDistanceMatrix(n_atoms,n_atoms)12:    **for** i←0 **to** n_atoms−1 **do**13:        **for** j←i+1 **to** n_atoms−1 **do**14:           distances[i,j]← Norm (c_alpha_atoms[i]−c_alpha_atoms[j])15:           distances[j,i]←distances[i,j]16:        **end for**17:    **end for**18:    threshold←8.0                                                                    ▹ hyperparameter19:    contact_map←filterContactMap(distances≤threshold,1,0)20:    pca←PCA(n_components=0.99)21:    contact_map_1d←pca.fit_transform(contact_map)22:    contact_map_1d←contact_map_1d.flatten()23:    **return** contact_map_1d24:**end function**

The technique incorporates principal component analysis (PCA) into its workflow to reduce the dimensionality of the contact map while maintaining its essential structural information. PCA is a powerful dimensionality reduction method that helps extract important information from the contact map. After it is created, the contact map is transformed using PCA, which consciously reduces its dimensionality. Principal components, or linear combinations of the features of the original contact map that capture the largest variance in the data, are extracted as part of this transformation. PCA allows for dimensionality reduction without sacrificing the critical structural properties of the contact map by keeping the elements that capture the greatest amount of variance. Next, the converted contact map is reshaped or flattened into a one-dimensional array, yielding what is known as the contact map-based embedding. It is a concise yet instructive depiction of the structural characteristics of the protein that captures the important spatial interactions between its constituent C-alpha atoms. This novel algorithmic method offers a potent way to represent proteins according to their contact maps. The resulting contact map-based embeddings provide vital insights into the intricate structural features of proteins by condensing and interpreting extensive structural information into a simplified form. These embeddings provide the foundation for classification and downstream analytic tasks, enabling researchers to better understand protein structures and build complex models for a range of biological and computational applications. The contact map-based embedding is concatenated with a large language model (LLM)-based embedding method such as SeqVec [37] and a feature engineering-based method such as Spike2Vec [47], which are designed for sequence-only embedding (without considering structural information), to enhance the performance of the final embedding representation for the proteins. The details for SeqVec and Spike2Vec are given in Section 4.3.

## 4. Experimental Setup

In this section, we present the dataset overview, machine learning classifiers, and evaluation metrics detail. The experiments were conducted on an Ubuntu 64-bit OS (16.04.7 LTS Xenial Xerus) system with an Intel(R) Xeon(R) CPU E7-4850 v4 @ 2.10GHz processor and 3023 GB of memory. We use a 70–30% train–test split of data, with 10% of the training data reserved for hyperparameter tuning. The experiments were repeated five times using random splits to ensure reliable and consistent results, and the average and standard deviation of the outcomes were evaluated. For classification, we use SVM, naive Bayes (NB), multi-layer perceptron (MLP), KNN, random forest (RF), logistic regression (LR), and decision tree (DT). For evaluation, we use average accuracy, precision, recall, weighted F1, macro F1, ROC AUC, and training runtime. In cases where the metrics were originally designed for binary classification, we utilized the one-vs.-rest approach to adapt them for multi-class classification scenarios.

In our study, we use well-established two benchmark datasets. The preprocessed data, necessary for reproducing our results along with code, is publicly available online (https://github.com/pchourasia1/PDB_Plus_LLM_Contact_Map, accessed on 20 December 2023). We use the following datasets:

### 4.1. STCRDAB

The STCRDAB (Structural T-Cell Receptor Database) [53] dataset is a meticulously curated collection of T-cell receptor structural data sourced from the Protein Data Bank (PDB). It consists of a total of 512 protein structures, downloaded as of 27 May 2021. For our experiment, we selected 480 PDB files from this dataset (after pre-processing), where the protein structures are classified into two classes: “Humans” (total 325 PDB files) and “Mouse” (total 155 PDB files), also shown in Table 1. Thus, the classification problem is binary. The minimum, maximum, and average lengths of sequences extracted from PDB files in the STCRDAB dataset are 109, 5415, and 1074.38, respectively.

### 4.2. PDB Bind

For the PDB Bind dataset, we obtained version 2020 [54] from the official source. The initial dataset consisted of a total of 14,127 PDB structures. After preprocessing, we selected 3792 structures for our analysis. The target labels used in this dataset correspond to the protein names, as presented in Table 2. The minimum, maximum, and average lengths of sequences extracted from PDB files in the PDB Bind dataset are 33, 3292, and 403.60, respectively.

### 4.3. Baseline Models

We use the Spike2Vec, SeqVec, and Unsupervised Protein Embeddings (UPE) as baseline models. The details for the baseline models are below:

#### 4.3.1. Spike2Vec [47]

It extracts features from protein sequences using the concept of *k*-mers, which represents a contiguous substring of length *k* within a sequence. For this study, we used k=3 to obtain the embeddings, chosen through standard validation. This choice ensures computational efficiency and captures sequence characteristics effectively. The length of the Spike2Vec-based embedding depends on the number of unique amino acids, denoted as *ACDEFGHIKLMNPQRSTVWXY*. The embedding length is ${\left|\Sigma \right|}^{k}$, providing a representation that encompasses diverse amino acid properties, making it a promising tool for computational biology applications.

#### 4.3.2. SeqVec [37]

It represents protein sequences as continuous vectors using the ELMO (Embeddings from Language Models) language model [55]. ELMO leverages the biophysical characteristics derived from unlabeled data from UniRef50 to generate embeddings (hence considered a large language model-based approach). This process, known as SeqVec (Sequence-to-Vector), assigns embeddings to individual words while taking into account their contextual information. By employing ELMO, it effectively captures the complex properties and relationships within protein sequences, enabling more comprehensive analysis and interpretation.

#### 4.3.3. Unsupervised Protein Embeddings (UPE) [56]

It is an unsupervised deep learning approach for generating protein embeddings that considers both sequence and structural information. It uses a technique from [37] to generate initial embeddings from sequences. For structural features, it utilizes one-hot encoding of secondary structure angles derived from the protein’s 3D structure. The final protein representation is obtained by combining sequence and structural features. Unlike our proposed contact map-based approach, their method does not use one-hot encoding for embedding 3D structural information due to issues with dimensionality and information preservation [36].

## 5. Results and Discussion

In this section, we present the results of our proposed method under various settings and compare its performance with baseline approaches on two datasets using different evaluation metrics.

The classification results are summarized in Table 3 and Table 4. When considering sequence-only embedding methods, we observe that the majority of cases achieve a predictive performance of over 90% for both datasets, surpassing the results obtained with structure-only approaches. However, the runtime is reduced significantly when SeqVec + Spike2Vec are used together for both databases. But accuracy deteriorates significantly, which can be seen in Table 3. The same can be seen in the case of PDB Bind data in Table 4. This can be attributed to the fact that the functional regions of protein sequences are often more conserved across different proteins compared to their 3D structures, making them easier to identify and predict. Consequently, sequence-based models demonstrate greater effectiveness in protein function prediction and classification. Additionally, sequence-based models are simpler than 3D structure-based autoencoder models, as they do not need to account for the complexities of protein folding and interactions. This simplicity makes them more manageable to train and interpret, resulting in improved performance. Overall, our findings indicate that the Spike2Vec embedding with the LR classifier outperforms all other classifiers for the STCRDAB dataset. A similar trend is observed for the PDB Bind dataset. The superior performance of Spike2Vec can be attributed to the fact that SeqVec, the large language model (LLM), is trained on diverse protein sequences from the UniRef50 dataset, which may not effectively generalize to the sequences extracted from the PDB files in our benchmark datasets. It is worth noting that the PDB Bind dataset is widely acknowledged as a challenging benchmark for structure-based prediction methods. Consequently, we observed a relatively lower predictive performance when using structure-based embeddings in this dataset.

When we combine sequences and structure embeddings (i.e., contact map + Spike2Vec, contact map + SeqVec, and contact map + SeqVec + Spike2Vec), we can observe that the predictive performance for all classifiers increases. Eventually, the contact map + SeqVec + Spike2Vec outperforms all other methods. This is because when combining structure and sequence embeddings, we are incorporating more information about the protein and its environment, which can help improve the accuracy. The sequence embeddings capture the amino acid composition and ordering of the protein sequence, while the structure-based embeddings capture the 3D spatial arrangement of the atoms in the protein structure. By combining these two sources of information, we can leverage the strengths of both methods and obtain a more comprehensive representation of the protein. Moreover, the proposed sequence + structural method outperforms the baseline UPE [56] for all evaluation metrics and both datasets.

Our study demonstrates that combining structure and sequence information in protein analysis improves the predictive performance compared to using either information alone. While the proposed method achieves a reasonable performance when considering structure information alone, a higher performance is observed when using sequence information alone. This is likely due to the conservation of functional regions across different proteins in the sequence, making them easier to identify and predict. By combining both structure and sequence information, we obtain a comprehensive representation of the protein, considering both structural features and sequence variations. This combination leads to almost perfect predictive performance, highlighting the complementary nature of structure and sequence-based embeddings in protein classification. Incorporating both types of information allows for a holistic understanding of protein function and interactions, resulting in improved classification outcomes.

We ensured the reliability and consistency of the classification result through a statistical analysis using *p*-values. The analysis is based on the average and standard deviation values of five experimental runs for both datasets. The *p*-values determined the statistical significance of comparisons between the proposed model and baselines. The comparisons had *p*-values below 0.05, indicating statistically significant performance differences. However, for the training runtime metric, some *p*-values exceeded 0.05 due to greater variability in runtime values. Factors like processor performance and active processes during training can affect the runtime variability. It is important to note that our analysis focused primarily on predictive performance evaluation metrics rather than training runtime.

## 6. Conclusions

This research explores the complex interactions between protein sequences, with a focus on using 3D structural data and large language model (LLM) techniques to transform protein classification. Our thorough analysis highlights the significant synergy that results from combining these disparate data sources, demonstrating the unmatched complementary nature. Specifically, our results demonstrate the impressive performance gain achieved with a hybrid method that outperforms the effectiveness of single data modalities. Our experimental results provide empirical data that are notably different from the performance differences that are seen when using 3D structure information exclusively as opposed to using protein sequences alone. Its significantly lower performance in the classification framework is illustrated by our empirical results, which also highlight the drawbacks of depending only on structural information. Conversely, leveraging protein sequences in isolation exhibits a notable enhancement in performance metrics, showcasing its efficacy as a standalone information source. Our study shows that combining structure and sequence information in protein analysis improves predictive performance compared to using either information alone.

This research delves into the intricate interplay between protein sequences, particularly leveraging large language model (LLM) approaches and 3D structural information to revolutionize protein classification. Our comprehensive investigation underscores the profound synergy achieved by integrating these distinct information sources, showcasing the unparalleled complementarity that exists between them. Notably, our findings illuminate the remarkable performance boost attained through a combined approach, surpassing the efficacy of individual data modalities. Empirical evidence from our experiments starkly contrasts the performance disparities observed when relying solely on 3D structural information versus leveraging protein sequences in isolation. Our empirical findings underscore the limitations associated with relying solely on 3D structural information, demonstrating its relatively diminished performance within the classification framework. Conversely, leveraging protein sequences in isolation exhibits a notable enhancement in performance metrics, showcasing its efficacy as a standalone information source.

In the future, we will be at the forefront of creating complex deep learning systems that are designed to seamlessly combine and utilize structural and sequence data. The goal of this project is to develop a state-of-the-art model that optimizes classification accuracy and precision by maximizing the synergies between various modalities. In addition, we investigate graph-based models, which have the potential to transform the way complex 3D structural data are embedded and used in classification algorithms. The plan for our upcoming projects includes a thorough assessment approach that goes beyond the limitations of our available datasets. It might be useful to test our suggested model on a variety of datasets to obtain a thorough evaluation of their interpretability, robustness, and scalability. This rigorous evaluation strategy intends to validate the generalizability and applicability of our methodologies beyond specific datasets, thereby fortifying the credibility and utility of our approach within the broader scientific community.

## Figures and Tables

**Table 1:** Class/target label statistics for STCRDAB dataset.

Species	Count
Human	325
Mouse	155
Total	480

**Table 2:** Class/target label statistics for PDB Bind dataset.

Target Name	Count	Target Name	Count
SERINE/THREONINE—PROTEIN	404	PROTEIN	138
TYROSINE—PROTEIN	381	E3	138
MITOGEN—ACTIVATED	325	CYCLIN-DEPENDENT	128
BETA—SECRETASE	299	GLUTAMATE	112
BETA—LACTAMASE	220	DUAL	111
BROMODOMAIN—CONTAINING	174	HEAT	110
HIV-1	164	PROTEASOME	110
CARBONIC	159	TANKYRASE-2	108
CELL	157	LYSINE-SPECIFIC	105
GLYCOGEN	144	DNA	104
PHOSPHATIDYLINOSITOL-45-BISPHOSPHATE	100	COAGULATION	101
		Total	3792

**Table 3:** Average classification results (of 5 runs) for different methods and STCRDAB datasets. The best values are shown in bold. The up arrow in the metric indicates a higher value is better while down arrow indicates a lower value is better.

Category	Embedding	Algo.	Acc. ↑	Prec. ↑	Recall ↑	F1 (Weig.) ↑	F1 (Macro) ↑	ROC-AUC ↑	Train Time (s) ↓
Sequence Only (Baselines)	Spike2Vec [47]	SVM	0.976	0.977	0.976	0.976	0.972	0.967	1.824
		NB	0.978	0.978	0.978	0.978	0.974	0.967	0.189
		MLP	0.983	0.984	0.983	0.983	0.981	0.982	5.145
		KNN	0.963	0.963	0.963	0.962	0.956	0.948	0.087
		RF	0.975	0.975	0.975	0.975	0.971	0.967	0.462
		LR	0.988	0.988	0.988	0.987	0.985	0.986	0.119
		DT	0.957	0.957	0.957	0.957	0.950	0.948	0.204
	SeqVec [37]	SVM	0.794	0.795	0.794	0.783	0.750	0.737	0.025
		NB	0.743	0.741	0.743	0.739	0.708	0.702	0.004
		MLP	0.726	0.734	0.726	0.727	0.700	0.705	3.297
		KNN	0.829	0.830	0.829	0.820	0.793	0.778	0.047
		RF	0.812	0.854	0.812	0.788	0.747	0.726	0.590
		LR	0.886	0.886	0.886	0.884	0.871	0.864	0.030
		DT	0.790	0.787	0.790	0.786	0.757	0.749	0.114
	SeqVec + Spike2Vec	SVM	0.882	0.886	0.882	0.876	0.857	0.840	0.051
		NB	0.829	0.828	0.829	0.827	0.803	0.795	**0.002**
		MLP	0.767	0.769	0.767	0.768	0.739	0.741	0.651
		KNN	0.926	0.933	0.926	0.924	0.912	0.893	0.033
		RF	0.913	0.917	0.913	0.910	0.895	0.877	0.331
		LR	0.982	0.982	0.982	0.982	0.980	0.980	0.019
		DT	0.897	0.897	0.897	0.897	0.884	0.881	0.053
Sequence + Structure (Baseline)	UPE [56]	SVM	0.916	0.989	0.916	0.988	0.909	0.907	0.961
		NB	0.897	0.908	0.897	0.895	0.896	0.911	0.975
		MLP	0.915	0.929	0.915	0.928	0.983	0.971	1.097
		KNN	0.921	0.928	0.921	0.929	0.981	0.979	0.452
		RF	0.894	0.885	0.894	0.892	0.881	0.893	0.813
		LR	0.957	0.942	0.957	0.954	0.975	0.963	0.128
		DT	0.901	0.899	0.901	0.900	0.921	0.943	0.042
Structure Only (ours)	Contact Map	SVM	0.569	0.556	0.569	0.560	0.514	0.517	0.040
		NB	0.639	0.644	0.639	0.624	0.584	0.591	0.007
		MLP	0.563	0.563	0.563	0.544	0.498	0.515	4.255
		KNN	0.621	0.554	0.621	0.537	0.453	0.509	0.048
		RF	0.646	0.554	0.646	0.511	0.403	0.506	0.747
		LR	0.664	0.653	0.664	0.648	0.605	0.603	0.037
		DT	0.579	0.572	0.579	0.573	0.531	0.532	0.207
Sequence + Structure (ours)	Contact Map + Spike2Vec	SVM	0.789	0.805	0.789	0.769	0.729	0.713	0.152
		NB	0.847	0.853	0.847	0.843	0.821	0.810	0.008
		MLP	0.614	0.619	0.614	0.603	0.552	0.559	18.721
		KNN	0.939	0.945	0.939	0.937	0.928	0.913	0.283
		RF	0.924	0.932	0.924	0.921	0.910	0.891	2.191
		LR	0.981	0.981	0.981	0.981	0.978	0.978	0.243
		DT	0.913	0.915	0.913	0.913	0.902	0.905	0.330
Sequence + Structure (ours)	Contact Map + SeqVec	SVM	0.840	0.869	0.840	0.819	0.768	0.737	0.061
		NB	0.800	0.798	0.800	0.784	0.730	0.710	0.007
		MLP	0.690	0.697	0.690	0.688	0.632	0.639	3.287
		KNN	0.844	0.844	0.844	0.843	0.813	0.810	0.043
		RF	0.824	0.850	0.824	0.800	0.743	0.714	0.779
		LR	0.879	0.881	0.879	0.880	0.858	0.861	0.077
		DT	0.799	0.801	0.799	0.799	0.764	0.766	0.286
Sequence + Structure (ours)	Contact Map + SeqVec + Spike2Vec	SVM	**0.991**	**0.990**	**0.991**	**0.990**	**0.988**	**0.985**	81.170
		NB	0.988	0.988	0.988	0.987	0.985	0.982	0.320
		MLP	0.988	0.988	0.988	0.988	0.985	0.988	11.968
		KNN	0.940	0.942	0.940	0.939	0.924	0.909	0.330
		RF	0.979	0.980	0.979	0.979	0.974	0.970	0.745
		LR	0.986	0.986	0.986	0.986	0.983	0.980	0.889
		DT	0.956	0.957	0.956	0.956	0.948	0.951	0.410

**Table 4:** Average classification results (of 5 runs) for different methods using PDB Bind datasets. The best values are shown in bold. The up arrow in the metric indicates a higher value is better while down arrow indicates a lower value is better.

Category	Embedding	Algo.	Acc. ↑	Prec. ↑	Recall ↑	F1 (Weig.) ↑	F1 (Macro) ↑	ROC-AUC ↑	Train Time (s) ↓
Sequence Only (Baselines)	Spike2Vec [47]	SVM	0.960	0.965	0.960	0.961	0.954	0.975	263.112
		NB	0.943	0.956	0.943	0.944	0.931	0.964	8.230
		MLP	0.934	0.939	0.934	0.934	0.919	0.958	85.427
		KNN	0.896	0.954	0.896	0.910	0.897	0.941	1.961
		RF	0.960	0.966	0.960	0.961	0.954	0.975	6.888
		LR	0.966	0.967	0.966	0.966	0.959	0.978	8.471
		DT	0.939	0.942	0.939	0.939	0.929	0.962	4.682
	SeqVec [37]	SVM	0.845	0.881	0.845	0.846	0.857	0.909	4.124
		NB	0.301	0.550	0.301	0.299	0.300	0.632	0.209
		MLP	0.745	0.756	0.745	0.741	0.735	0.865	32.370
		KNN	0.828	0.849	0.828	0.830	0.817	0.901	0.311
		RF	0.822	0.876	0.822	0.829	0.844	0.898	7.645
		LR	0.874	0.880	0.874	0.874	0.870	0.927	19.388
		DT	0.783	0.782	0.783	0.781	0.782	0.887	7.134
	SeqVec + Spike2Vec	SVM	0.883	0.905	0.883	0.882	0.884	0.925	12.571
		NB	0.688	0.765	0.688	0.703	0.692	0.841	**0.136**
		MLP	0.757	0.768	0.757	0.754	0.747	0.873	9.640
		KNN	0.919	0.942	0.919	0.924	0.912	0.954	2.412
		RF	0.935	0.943	0.935	0.937	0.929	0.961	6.024
		LR	0.958	0.962	0.958	0.959	0.951	0.974	22.074
		DT	0.878	0.881	0.878	0.878	0.865	0.930	2.765
Sequence + Structure (Baseline)	UPE [56]	SVM	0.891	0.912	0.891	0.942	0.929	0.899	6.581
		NB	0.922	0.941	0.922	0.918	0.919	0.896	1.675
		MLP	0.963	0.922	0.963	0.921	0.905	0.896	4.254
		KNN	0.959	0.923	0.959	0.949	0.938	0.893	0.234
		RF	0.921	0.944	0.921	0.932	0.928	0.948	4.563
		LR	0.954	0.925	0.954	0.930	0.929	0.965	9.753
		DT	0.939	0.928	0.939	0.935	0.912	0.945	0.973
Structure Only (ours)	Contact Map	SVM	0.585	0.823	0.585	0.627	0.665	0.779	25.248
		NB	0.352	0.440	0.352	0.345	0.325	0.657	0.947
		MLP	0.502	0.570	0.502	0.505	0.510	0.748	80.370
		KNN	0.571	0.706	0.571	0.599	0.574	0.760	0.482
		RF	0.690	0.759	0.690	0.694	0.702	0.821	19.493
		LR	0.712	0.726	0.712	0.713	0.699	0.840	151.352
		DT	0.578	0.586	0.578	0.579	0.571	0.777	17.549
Sequence + Structure (ours)	Contact Map + Spike2Vec	SVM	0.678	0.827	0.678	0.706	0.731	0.821	17.635
		NB	0.426	0.501	0.426	0.431	0.409	0.690	0.575
		MLP	0.535	0.588	0.535	0.535	0.536	0.767	105.944
		KNN	0.593	0.769	0.593	0.637	0.634	0.784	0.475
		RF	0.841	0.866	0.841	0.841	0.844	0.904	14.192
		LR	0.918	0.923	0.918	0.919	0.908	0.952	138.618
		DT	0.775	0.779	0.775	0.774	0.766	0.880	12.891
Sequence + Structure (ours)	Contact Map + SeqVec	SVM	0.802	0.870	0.802	0.807	0.810	0.877	34.657
		NB	0.459	0.533	0.459	0.451	0.443	0.715	1.630
		MLP	0.553	0.598	0.553	0.554	0.558	0.777	64.202
		KNN	0.552	0.717	0.552	0.589	0.573	0.754	0.465
		RF	0.798	0.853	0.798	0.806	0.820	0.885	19.770
		LR	0.804	0.809	0.804	0.803	0.802	0.894	196.321
		DT	0.714	0.717	0.714	0.713	0.710	0.850	25.274
Sequence + Structure (ours)	Contact Map + SeqVec + Spike2Vec	SVM	0.677	0.816	0.677	0.701	0.728	0.818	17.343
		NB	0.430	0.528	0.430	0.441	0.425	0.697	0.498
		MLP	0.524	0.568	0.524	0.524	0.530	0.761	120.589
		KNN	0.671	0.726	0.671	0.682	0.686	0.828	0.429
		RF	0.839	0.860	0.839	0.839	0.844	0.904	14.844
		LR	**0.968**	**0.972**	**0.968**	**0.969**	**0.966**	**0.980**	134.948
		DT	0.764	0.772	0.764	0.766	0.762	0.875	12.175

## Data Availability

The data are available at: https://github.com/pchourasia1/PDB_Plus_LLM_Contact_Map.

## References

[1] AlQuraishi M. (2021). Machine learning in protein structure prediction. Curr. Opin. Chem. Biol..

[2] Kubinyi H. (1998). Structure-based design of enzyme inhibitors and receptor ligands. Curr. Opin. Drug Discov. Dev..

[3] Zou L., Chen L., Lu Y. Top-k subgraph matching query in a large graph. Proceedings of the ACM First Ph.D. Workshop in CIKM.

[4] Licheri N., Amparone E., Bonnici V., Giugno R., Beccuti M. An Entropy Heuristic to Optimize Decision Diagrams for Index-driven Search in Biological Graph Databases. Proceedings of the CIKM Workshops.

[5] Batool M., Ahmad B., Choi S. (2019). A structure-based drug discovery paradigm. Int. J. Mol. Sci..

[6] Burley S.K., Berman H.M., Kleywegt G.J., Markley J.L., Nakamura H., Velankar S. (2017). Protein Data Bank (PDB): The single global macromolecular structure archive. Protein Crystallography: Methods and Protocols.

[7] Kmiecik S., Gront D., Kolinski M., Wieteska L., Dawid A.E., Kolinski A. (2016). Coarse-grained protein models and their applications. Chem. Rev..

[8] Schmidt T., Bergner A., Schwede T. (2014). Modelling three-dimensional protein structures for applications in drug design. Drug Discov. Today.

[9] Lounnas V., Ritschel T., Kelder J., McGuire R., Bywater R.P., Foloppe N. (2013). Current progress in structure-based rational drug design marks a new mindset in drug discovery. Comput. Struct. Biotechnol. J..

[10] De Lucrezia D., Slanzi D., Poli I., Polticelli F., Minervini G. (2012). Do natural proteins differ from random sequences polypeptides? Natural vs. random proteins classification using an evolutionary neural network. PLoS ONE.

[11] Clark W.T., Radivojac P. (2011). Analysis of protein function and its prediction from amino acid sequence. Proteins Struct. Funct. Bioinform..

[12] Radivojac P., Clark W.T., Oron T.R., Schnoes A.M., Wittkop T., Sokolov A., Graim K., Funk C., Verspoor K., Ben-Hur A. (2013). A large-scale evaluation of computational protein function prediction. Nat. Methods.

[13] Bailey T.L., Williams N., Misleh C., Li W.W. (2006). MEME: Discovering and analyzing DNA and protein sequence motifs. Nucleic Acids Res..

[14] Rives A., Meier J., Sercu T., Goyal S., Lin Z., Liu J., Guo D., Ott M., Zitnick C.L., Ma J. (2021). Biological structure and function emerge from scaling unsupervised learning to 250 million protein sequences. Proc. Natl. Acad. Sci. USA.

[15] Cavasotto C.N., Phatak S.S. (2009). Homology modeling in drug discovery: Current trends and applications. Drug Discov. Today.

[16] Li H., Homer N. (2010). A survey of sequence alignment algorithms for next-generation sequencing. Brief. Bioinform..

[17] Amitai G., Shemesh A., Sitbon E., Shklar M., Netanely D., Venger I., Pietrokovski S. (2004). Network analysis of protein structures identifies functional residues. J. Mol. Biol..

[18] Jing B., Eismann S., Suriana P., Townshend R.J., Dror R. (2020). Learning from protein structure with geometric vector perceptrons. arXiv.

[19] Haas J., Roth S., Arnold K., Kiefer F., Schmidt T., Bordoli L., Schwede T. (2013). The Protein Model Portal—a comprehensive resource for protein structure and model information. Database.

[20] Yan T.C., Yue Z.X., Xu H.Q., Liu Y.H., Hong Y.F., Chen G.X., Tao L., Xie T. (2022). A systematic review of state-of-the-art strategies for machine learning-based protein function prediction. Comput. Biol. Med..

[21] Bonetta R., Valentino G. (2020). Machine learning techniques for protein function prediction. Proteins Struct. Funct. Bioinform..

[22] Liu X. (2017). Deep recurrent neural network for protein function prediction from sequence. arXiv.

[23] Kuhlman B., Bradley P. (2019). Advances in protein structure prediction and design. Nat. Rev. Mol. Cell Biol..

[24] Madani A., Krause B., Greene E.R., Subramanian S., Mohr B.P., Holton J.M., Olmos Jr J.L., Xiong C., Sun Z.Z., Socher R. (2023). Large language models generate functional protein sequences across diverse families. Nature Biotechnol..

[25] Quintana F., Treangen T., Kavraki L. Leveraging Large Language Models for Predicting Microbial Virulence from Protein Structure and Sequence. Proceedings of the 14th ACM International Conference on Bioinformatics, Computational Biology, and Health Informatics.

[26] Lin Z., Akin H., Rao R., Hie B., Zhu Z., Lu W., Smetanin N., Verkuil R., Kabeli O., Shmueli Y. (2023). Evolutionary-scale prediction of atomic-level protein structure with a language model. Science.

[27] Ofer D., Brandes N., Linial M. (2021). The language of proteins: NLP, machine learning & protein sequences. Comput. Struct. Biotechnol. J..

[28] Lin Z., Akin H., Rao R., Hie B., Zhu Z., Lu W., dos Santos Costa A., Fazel-Zarandi M., Sercu T., Candido S. (2022). Language models of protein sequences at the scale of evolution enable accurate structure prediction. bioRxiv.

[29] Forslund K., Sonnhammer E.L. (2008). Predicting protein function from domain content. Bioinformatics.

[30] Pan X., Shen H.B. (2017). RNA-protein binding motifs mining with a new hybrid deep learning based cross-domain knowledge integration approach. BMC Bioinform..

[31] Klein P., Delisi C. (1986). Prediction of protein structural class from the amino acid sequence. Biopolym. Orig. Res. Biomol..

[32] Vinga S., Gouveia-Oliveira R., Almeida J.S. (2004). Comparative evaluation of word composition distances for the recognition of SCOP relationships. Bioinformatics.

[33] Ie E., Weston J., Noble W.S., Leslie C. Multi-class protein fold recognition using adaptive codes. Proceedings of the International Conference on Machine Learning.

[34] Shamim M.T.A., Anwaruddin M., Nagarajaram H.A. (2007). Support vector machine-based classification of protein folds using the structural properties of amino acid residues and amino acid residue pairs. Bioinformatics.

[35] Kuzmin K., Adeniyi A.E., DaSouza A.K., Lim D., Nguyen H., Molina N.R., Xiong L., Weber I.T., Harrison R.W. (2020). Machine learning methods accurately predict host specificity of coronaviruses based on spike sequences alone. Biochem. Biophys. Res. Commun..

[36] Ali S., Sahoo B., Ullah N., Zelikovskiy A., Patterson M., Khan I. A k-mer based approach for SARS-CoV-2 variant identification. Proceedings of the International Symposium on Bioinformatics Research and Applications.

[37] Heinzinger M., Elnaggar A., Wang Y., Dallago C., Nechaev D., Matthes F., Rost B. (2019). Modeling aspects of the language of life through transfer-learning protein sequences. BMC Bioinform..

[38] Brandes N., Ofer D., Peleg Y., Rappoport N., Linial M. (2022). ProteinBERT: A universal deep-learning model of protein sequence and function. Bioinformatics.

[39] Sofi M.A., Wani M.A. Improving Prediction of Protein Secondary Structures using Attention-enhanced Deep Neural Networks. Proceedings of the 2022 9th International Conference on Computing for Sustainable Global Development.

[40] Buchan D.W., Jones D.T. (2019). The PSIPRED protein analysis workbench: 20 years on. Nucleic Acids Res..

[41] Rozemberczki B., Gogleva A., Nilsson S., Edwards G., Nikolov A., Papa E. MOOMIN: Deep Molecular Omics Network for Anti-Cancer Drug Combination Therapy. Proceedings of the International Conference on Information & Knowledge Management (CIKM).

[42] Apeltsin L., Morris J.H., Babbitt P.C., Ferrin T.E. (2011). Improving the quality of protein similarity network clustering algorithms using the network edge weight distribution. Bioinformatics.

[43] Altschul S.F., Madden T.L., Schäffer A.A., Zhang J., Zhang Z., Miller W., Lipman D.J. (1997). Gapped BLAST and PSI-BLAST: A new generation of protein database search programs. Nucleic Acids Res..

[44] Altschul S.F., Gish W., Miller W., Myers E.W., Lipman D.J. (1990). Basic local alignment search tool. J. Mol. Biol..

[45] Altschul S.F., Wootton J.C., Gertz E.M., Agarwala R., Morgulis A., Schäffer A.A., Yu Y.K. (2005). Protein database searches using compositionally adjusted substitution matrices. FEBS J..

[46] Ali S., Bello B., Chourasia P., Punathil R.T., Zhou Y., Patterson M. (2022). PWM2Vec: An Efficient Embedding Approach for Viral Host Specification from Coronavirus Spike Sequences. Biology.

[47] Ali S., Patterson M. Spike2vec: An efficient and scalable embedding approach for COVID-19 spike sequences. Proceedings of the IEEE International Conference on Big Data (Big Data).

[48] Wood D., Salzberg S. (2014). Kraken: Ultrafast metagenomic sequence classification using exact alignments. Genome Biol..

[49] Girotto S., Pizzi C., Comin M. (2016). MetaProb: Accurate metagenomic reads binning based on probabilistic sequence signatures. Bioinformatics.

[50] De Oliveira S., Deane C. (2017). Co-evolution techniques are reshaping the way we do structural bioinformatics. F1000Research.

[51] Kuksa P., Khan I., Pavlovic V. Generalized Similarity Kernels for Efficient Sequence Classification. Proceedings of the SIAM International Conference on Data Mining (SDM).

[52] Kané H., Coulibali M.K., Ajanoh P., Abdallah A. (2019). Augmenting protein network embeddings with sequence information. bioRxiv.

[53] Leem J., de Oliveira S.H.P., Krawczyk K., Deane C.M. (2018). STCRDab: The structural T-cell receptor database. Nucleic Acids Res..

[54] Liu Z., Li Y., Han L., Li J., Liu J., Zhao Z., Nie W., Liu Y., Wang R. (2015). PDB-wide collection of binding data: Current status of the PDBbind database. Bioinformatics.

[55] Sarzynska-Wawer J., Wawer A., Pawlak A., Szymanowska J., Stefaniak I., Jarkiewicz M., Okruszek L. (2021). Detecting formal thought disorder by deep contextualized word representations. Psychiatry Res..

[56] Villegas-Morcillo A., Makrodimitris S., van Ham R.C., Gomez A.M., Sanchez V., Reinders M.J. (2021). Unsupervised protein embeddings outperform hand-crafted sequence and structure features at predicting molecular function. Bioinformatics.

